# Glucosylceramide Synthase Inhibition in Combination with Aripiprazole Sensitizes Hepatocellular Cancer Cells to Sorafenib and Doxorubicin

**DOI:** 10.3390/ijms26010304

**Published:** 2024-12-31

**Authors:** Richard Jennemann, Martina Volz, Roberto Carlos Frias-Soler, Almut Schulze, Karsten Richter, Sylvia Kaden, Roger Sandhoff

**Affiliations:** 1 Lipid Pathobiochemistry Group, German Cancer Research Center, Im Neuenheimer Feld 581, 69120 Heidelberg, Germanyr.sandhoff@dkfz.de (R.S.); 2 Division of Tumor Metabolism and Microenvironment, German Cancer Research Center (DKFZ) and DKFZ-ZMBH Alliance, Im Neuenheimer Feld 581, 69120 Heidelberg, Germany; 3 Institute of Pharmacy and Molecular Biotechnology (IPMB), University of Heidelberg, Im Neuenheimer Feld 364, 69120 Heidelberg, Germany; 4 Core Facility Electron Microscopy, German Cancer Research Center, Im Neuenheimer Feld 280, 69120 Heidelberg, Germany

**Keywords:** glucosylceramide synthase, anti-psychotic drugs, cationic amphiphilic drugs (CAD), aripiprazole, Genz, sorafenib, doxorubicin, mitochondrial function, lysosomal function, hepatocellular tumor spheroids

## Abstract

Hepatocellular carcinoma (*HCC*) is one of the leading causes of cancer deaths due to its late diagnosis and restricted therapeutic options. Therefore, the search for appropriate alternatives to commonly applied therapies remains an area of high clinical need. Here we investigated the therapeutic potential of the glucosylceramide synthase (GCS) inhibitor Genz-123346 and the cationic amphiphilic drug aripiprazole on the inhibition of Huh7 and Hepa 1-6 hepatocellular cancer cell and tumor microsphere growth. Single and combinatorial treatments with both drugs at 5 µM concentration led to efficient cell cycle arrest, reduced expression of cyclins A and E, increased lipid storage in lysosomal compartments, accompanied by increased uptake of lysotracker, and elevated expression of the autophagy marker Lc3 II. Both drugs affected mitochondrial function, indicated by altered mitotracker uptake and impaired mitochondrial respiration. Aripiprazole in monotherapy, or even more pronounced in combination with Genz, also potentiated the effect of the cytostatic drugs sorafenib and doxorubicin on tumor cell- and tumor spheroid-growth inhibition. Targeting GCS with Genz with the parallel application of cationic amphiphilic drugs such as aripiprazole in combination with cytostatic drugs may thus represent a potent therapeutic approach in the treatment of HCC and potentially other cancer types.

## 1. Introduction

Hepatocellular cancer (HCC), although relatively rare, represents one of the leading causes of cancer deaths worldwide [1]. Alcohol consumption, hepatitis virus infections [2], and metabolic dysfunction-associated fatty liver disease (MAFLD) causing non-alcoholic steatohepatitis (NASH) [3,4] represent major risk factors. With progressing disease stage, fibrosis and cirrhosis occur, accompanied by inflammation [5,6], which may induce and promote hepatocellular cancer [7,8]. Therapy is difficult since the majority of patients already present with advanced-stage cancer, limiting the options for successful treatment. Therefore, the identification of novel treatment options is of high clinical importance. Targeting glycosphingolipid (GSL) synthesis by blocking the rate-limiting enzyme glucosylceramide synthase (GCS) encoded by the gene UDP-glucose-ceramide-glucosyltransferase (*UGCG*) has been considered as an option in cancer therapy [9,10]. GSLs play an important role in cellular processes such as cell adhesion and signaling [11]. These lipids concentrate in membrane microdomains and can modify the activity of growth factor and cytokine receptors [11]. Several studies suggest that depletion of GSLs by targeting GCS may impede cancer cell growth, as shown by preclinical studies employing cell culture and xenograft models [12,13,14,15,16,17]. Alteration in GSL-composition may also influence cytokinesis, and deletion or reduction in these compounds was shown to affect cell division [18,19]. In line, we have previously shown that the direct targeting of *Ugcg* in murine hepatocytes or treatment with the specific GCS-inhibitor Genz-123346 led to the absence or reduction in GSLs and inhibited tumor growth in mouse models of chemically induced hepatocellular and colorectal cancer [20,21]. Although delayed tumorigenesis was observed in these mouse models, all animals ultimately developed tumors over time. Therefore, therapies solely targeting GCS appear to be of limited efficacy. However, it was shown that elevated GCS expression can induce drug resistance [22]. Therefore, lowering GCS may sensitize cancer cells to drug treatment [23,24,25].

Cationic amphiphilic drugs (CADs) have recently emerged as potential cancer therapies, as epidemiologic studies indicated potential cancer preventive effects of CADs [26,27,28,29,30,31]. Cancer patients diagnosed with non-localized disease who had received CAD-based tricyclic antidepressants and antihistamines before diagnosis have shown improved survival [26,27,28,29,30,31]. CADs are small molecules consisting of a hydrophobic tail ornamented with one or more aromatic rings and a hydrophilic amine, which can be protonated in lysosomes. CADs concentrate in lysosomes over time, and their positive charge interferes with the negative charging of intra-luminal vesicles, which is important for the proper function of intra-lysosomal enzymes. As a consequence, lysosomal degradation of sphingolipids and phospholipids is inhibited, leading to lipid accumulation [32]. Elevated lysosomal lipid storage may also lead to impaired lysosomal and mitochondrial function [33] and therefore reduce cancer cell growth [26,33,34,35,36,37,38]. As endocytosis and sphingolipid metabolism may be increased in cancer cells, corresponding treatment could affect them at lower concentrations and shorter exposure times than normal cells [39,40,41].

In the present study, we have investigated the influence of the GCS inhibition with Genz-123346 in combination with the psychotropic CAD aripiprazole on hepatocellular cancer cell growth, both as a single treatment or in combination with established chemotherapeutic agents. We show that Genz, either alone or in combination with aripiprazole, significantly enhanced the effect of sorafenib and doxorubicin, leading to a pronounced reduction in cancer cell growth in two-dimensional cultures and in tumor spheroids.

## 2. Results

### 2.1. Cell Cycle Arrest of Hepatocellular Cancer Cells upon Genz and Aripiprazole Treatment

**Figure 1:**
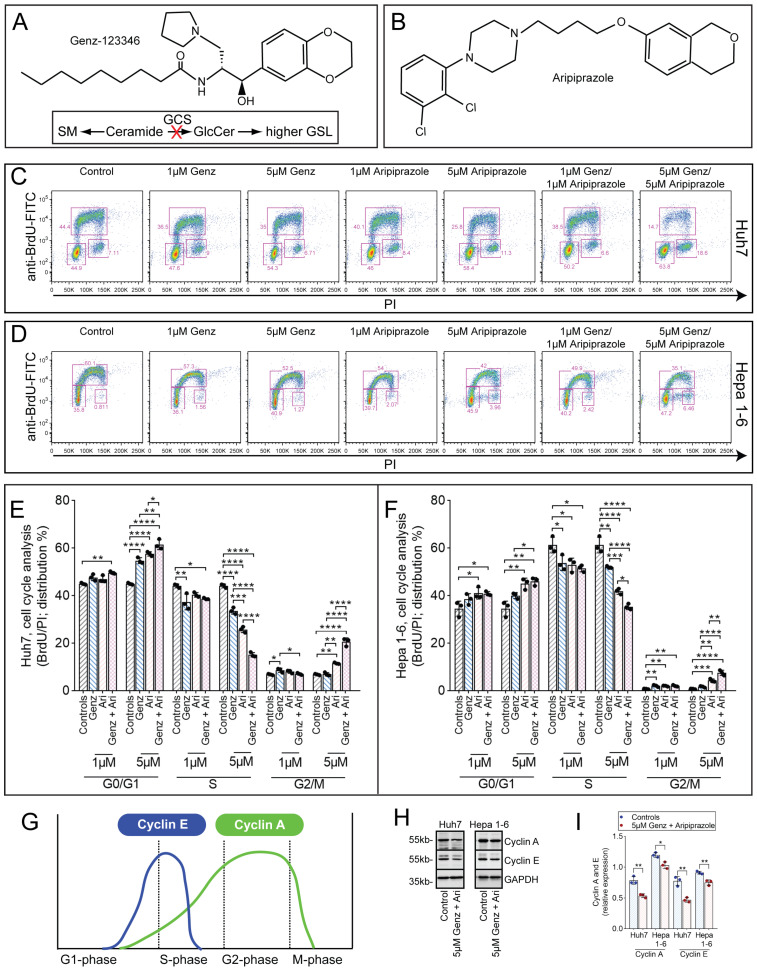
Genz and aripiprazole inhibit the cell cycle of cancer cells. (**A**,**B**) Chemical structures of Genz (**A**) and aripiprazole (Ari, (**B**)). Genz inhibits the basic step of the glycosphingolipid (GSL) biosynthesis pathway, the synthesis of glucosylceramide from ceramide and UDP-activated glucose, which is catalyzed by the enzyme glucosylceramide synthase (GCS) (**A**). (**C**–**F**) cell cycle analysis by BrdU/PI staining. Huh7 cells ((**C**) FACS images, (**E**) quantification) and Hepa 1-6 cells ((**D**) FACS images, (**F**) quantification); shown is one representative result out of two independent experiments; n = 3 for each condition; bar diagrams show mean values ± SD. Significances calculated by an one-way ANOVA test are: *, *p* ≤ 0.05; **, *p* < 0.01; ***, *p* < 0.001; ****, *p* < 0.0001; note: identical controls were plotted twice, one for each condition (**E**,**F**). (**G**) Scheme of the expression of cyclin E and A during cell cycle. (**H**) and ((**I**), quantification) Expression of cyclin A and E in Huh7 and Hepa 1-6 cells treated with 5 µM of Genz/5 µM aripiprazole; n = 3.

In order to establish the impact of the GCS-inhibitor Genz (Figure 1A) and the CAD aripiprazole (Figure 1B) on the cell cycle of hepatoma cells, Huh7 and Hepa 1-6 hepatocellular carcinoma cells were cultivated in the presence of the thymidine mimicry 5-Bromo-2′-desoxyuridine (BrdU). Application of Genz or aripiprazole in monotherapy at 1 µM concentrations induced a slight reduction in cells entering the S-phase both in Huh7 (Figure 1C,E) and Hepa 1-6 cells (Figure 1D,F). At 5 µM concentrations, both drugs caused a significant increase in the proportion of cells in GO/G1 phase (Figure 1E,F). Cells treated with 5 µM Genz in combination with 5 µM aripiprazole showed an even stronger reduction in the proportion of cells entering S-phase and an increased number of cells arrested in the G2/M phase (Figure 1C,D, right panel, and quantification in Figure 1E,F). G2/M arrest went along with an increased number of cells displaying a rounded morphology, indicating that these cells had not completed cytokinesis (Supplementary Figure S1). Protein levels of cyclin E and A, which are usually highly expressed in proliferative cells during S- or G2/M-phase (Figure 1G), were also significantly decreased (Figure 1H,I).

### 2.2. Genz Treatment Induces Apoptosis and Necrosis in Hepa 1-6 Cells

**Figure 2:**
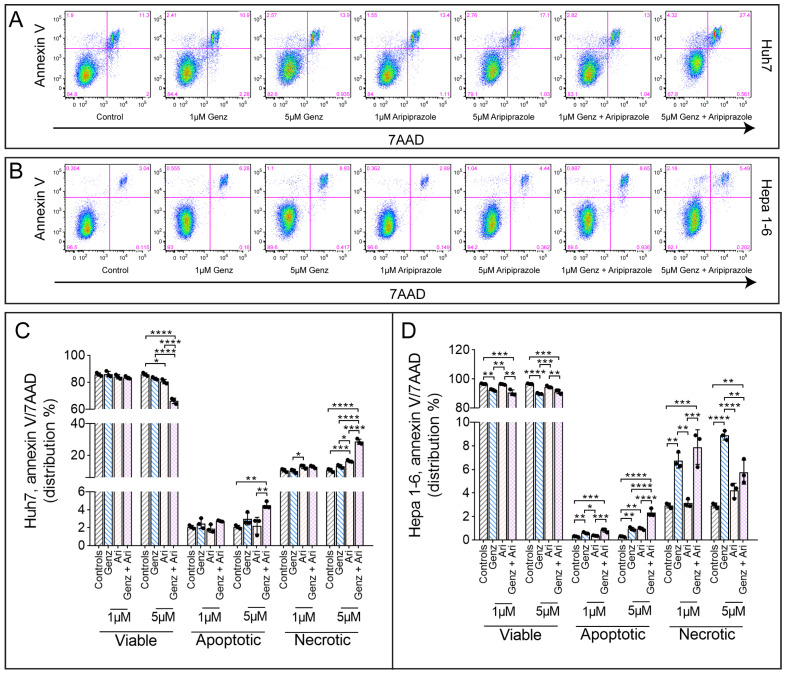
Aripiprazole treatment affects cell apoptosis and necrosis. (**A**–**D**) Annexin V/7AAD FACS staining of HCC cancer cells treated with different concentrations of Genz and aripiprazole (Ari) as indicated. (**A**) Representative FACS images from Huh7 cells and Hepa 1-6 cells (**B**). (**C**) Quantification of the FACS results obtained from Huh7 or (**D**) Hepa 1-6 cells in the presence of the drugs; shown is one representative experiment from three independent experiments; n = 3 for each condition; note: identical controls were plotted twice, one for each condition (**C**) and (**D**). Bar diagrams show mean values ± SD. Significances calculated with an one-way ANOVA test are: *, *p* ≤ 0.05; **, *p* < 0.01; ***, *p* < 0.001; ****, *p* < 0.0001.

Having demonstrated that Genz and aripiprazole treatment affected the cell cycle, we next asked whether these drugs could also increase the number of apoptotic or necrotic cells by performing annexin V/7AAD staining. Cultures of Huh7 cells treated with 5 µM Genz in combination with aripiprazole displayed markedly elevated numbers of annexin V^+^ apoptotic and annexin V^+^/7AAD^+^ necrotic cells, as compared to the untreated controls (Figure 2A,C). Remarkably, elevated numbers of apoptotic and necrotic cells were seen upon single-agent Genz treatment in the Hepa 1-6 cell line, whereas aripiprazole only marginally affected apoptosis and necrosis in these cells (Figure 2B,D).

### 2.3. Combined Treatment with 5 µM Each of Genz and Aripiprazole Enhances Lysotracker Uptake

**Figure 3:**
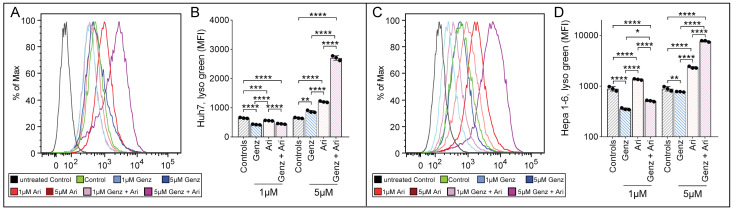
Aripiprazole enhances lysotracker incorporation into cancer cells. (**A**–**D**) Lysotracker uptake of HCC cancer cells in the presence of different concentrations of Genz and aripiprazole (Ari). (**A**) FACS images of Huh7 cells and (**B**) quantification of the medium fluorescence intensity (MFI). (**C**) FACS images of Hepa 1-6 cells and (**D**) quantification of the MFI; shown is one representative result out of two independent experiments; n = 3 for each condition; note: controls were plotted in duplicates, one for each condition (**B**) and (**D**). Bar diagrams show mean values ± SD. Significances calculated with an one-way ANOVA test are: *, *p* ≤ 0.05; **, *p* < 0.01; ***, *p* < 0.001; ****, *p* < 0.0001.

In order to test whether lysosomal function may have been affected by Genz or aripiprazole treatment, Huh7 and Hepa 1-6 cells were treated with the respective drugs and stained with lysotracker. The mean fluorescence intensity of lysotracker-stained cells increased upon aripiprazole application in both cell lines (Huh7, Figure 3A,B; Hepa 1-6, Figure 3C,D). In contrast, Genz treatment only caused a moderate effect on lysotracker incorporation. However, combined treatment with 5 µM of Genz with 5 µM of aripiprazole induced a strong increase in lysotracker staining in both cell lines.

### 2.4. Combined Treatment with 5 µM Each of Genz and Aripiprazole Elevates the Expression of the Autophagy Marker Lc3 II and Affects Lysosomal Ultrastructure

**Figure 4:**
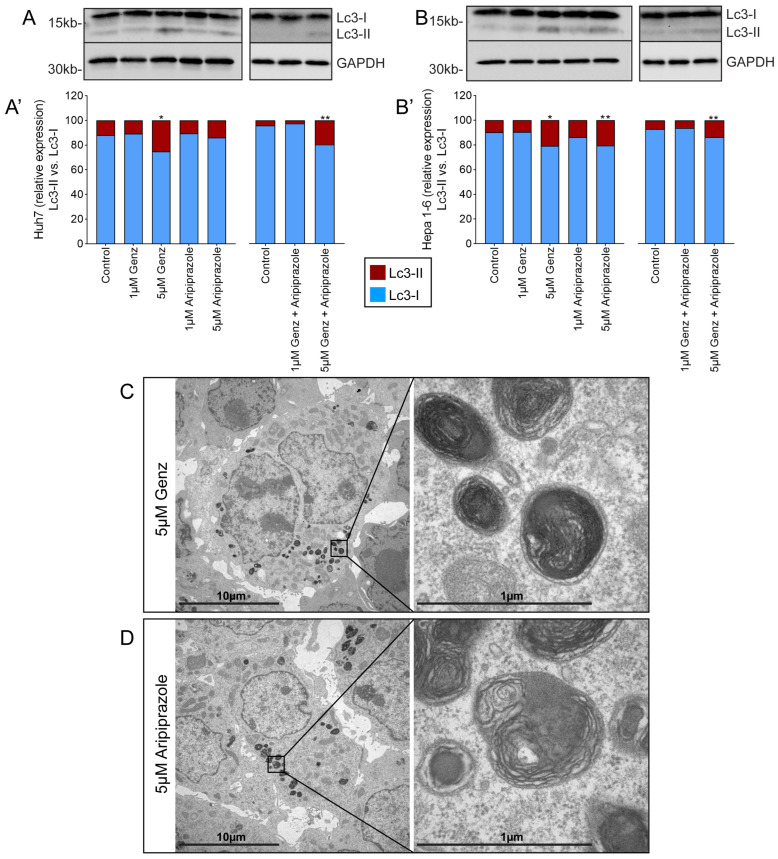
Elevated Genz and aripiprazole concentrations cause lipid storage in lysosomal compartments and enhance the expression of Lc3 II. Lc3 II expression of Huh7 ((**A**) western blot, (**A’**) quantification) and Hepa 1-6 cells ((**B**) western blot, (**B’**) quantification) treated with Genz and/or aripiprazole as indicated; n = 3 each. Bar diagrams show mean values. Significances calculated with an one-tailed *t*-test (controls vs. treated) are: *, *p* ≤ 0.05; **, *p* < 0.01. (**C**,**D**) Electron micrographs from Hepa 1-6 cells treated with either 5 µM Genz (**C**) or 5 µM aripiprazole (**D**); note the intense lipid staining of lamellar bodies within lysosomal compartments.

**Figure 5:**
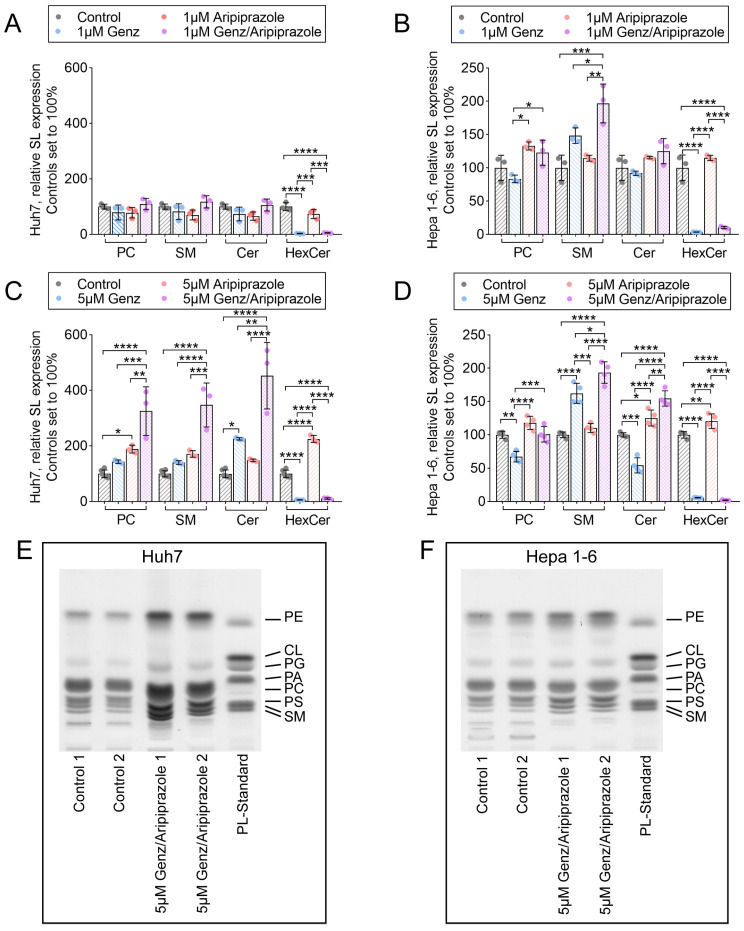
Treatment of Huh7 and Hepa 1-6 cells with 5 µM Genz and aripiprazole elevates cellular phospholipid and sphingomyelin (SM) content. (**A**–**D**) Relative expression of sphingolipids isolated from HCC cancer cells treated with Genz and aripiprazole. (**A**,**B**) UPLC-MS/MS analyses of Huh7 (**A**) and Hepa (1-6) cells (**B**) treated with 1 µM Genz or 1 µM aripiprazole and a combination of both drugs; n = 3 each. (**C**,**D**) UPLC-MS/MS analyses of Huh7 (**C**) and Hepa (1-6) cells (**D**) treated with 5 µM Genz or 5 µM aripiprazole and a combination of both drugs; n = 4 each. Bar diagrams show mean values ± SD. Significances calculated with an one-way ANOVA-test are: *, *p* ≤ 0.05; **, *p* < 0.01; ***, *p* < 0.001; ****, *p* < 0.0001. (**E**,**F**) Qualitative thin-layer chromatogram (TLC) of phospholipids isolated from Huh7 cells (**E**) and Hepa 1-6 cells (**F**) treated with 5 µM Genz/5 µM aripiprazole.

Alteration in the lysotracker staining due to drug treatment may indicate impaired lysosomal function and affect autophagy. Indeed, the expression of the autophagy marker protein Lc3 II was clearly elevated in Huh7 and Hepa 1-6 cells treated with 5 µM Genz, either alone or in combination with 5 µM aripiprazole (Figure 4A,B; Figure 4A’,B’). In addition, Hepa 1-6 cells showed elevated Lc3 II levels also after treatment with 5 µM of aripiprazole (Figure 4B,B’). Furthermore, ultrastructural analysis, exemplarily shown for Hepa 1-6 cells, revealed staining of multilamellar bodies, typically occurring due to lipid storage within lysosomes, upon treatment with 5 µM Genz (Figure 4C) or 5 µM aripiprazole (Figure 4D).

### 2.5. Sphingolipid and/or Phospholipid Concentrations Significantly Increase in Response to 5 µM Genz and Aripiprazole Application

In order to monitor alterations of cellular lipid content in hepatoma cells upon Genz and aripiprazole treatment, lipids were extracted and quantified by ultra-performance liquid chromatography-mass spectrometry (UPLC-MS/MS) and thin layer chromatography (TLC). UPLC-MS/MS analysis revealed that the relative amounts of sphingomyelin (SM), phosphatidylcholine (PC), and ceramides were not substantially altered in Huh7 cells treated with 1 µM Genz or 1 µM aripiprazole or a combination of both compounds as compared to non-treated controls (Figure 5A). However, in Huh7 cells incubated with 5 µM of Genz or 5 µM of aripiprazole, the concentrations of PC and SM significantly increased. Moreover, the combination of both drugs at this concentration led to an even higher induction of those lipids. In contrast, the relative abundance of GlcCer (HexCer) was almost completely depleted by the addition of the GCS-inhibitor Genz, whereas aripiprazole application only caused a slight increase in these lipids (Figure 5C).

SM levels increased in Hepa 1-6 cells treated with 1 µM Genz (Figure 5B), whereas PC and ceramides showed a surprising drop in abundance, particularly upon treatment with 5 µM Genz in these cells (Figure 5D). Aripiprazole application, on the other hand, increased levels of PC and SM in Hepa 1-6 cells (Figure 5B,D). Treatment of these cells with a combination of both drugs led to an increase in SM levels, as compared to single Genz or aripiprazole administration (Figure 5D). TLC detection of total lipid extracts of hepatoma cells confirmed the general influence of Genz/aripiprazole treatment on phospholipid and SM content in both cell lines (Figure 5E, Huh7, and 5F, Hepa 1-6). Similar effects of both drugs on lipid abundance were also observed in HepG2 cells treated with 5 µM Genz and aripiprazole (Supplementary Figure S2A,B).

### 2.6. Genz and Aripiprazole Treatment Affects Uptake of Mitotracker Green and Red

**Figure 6:**
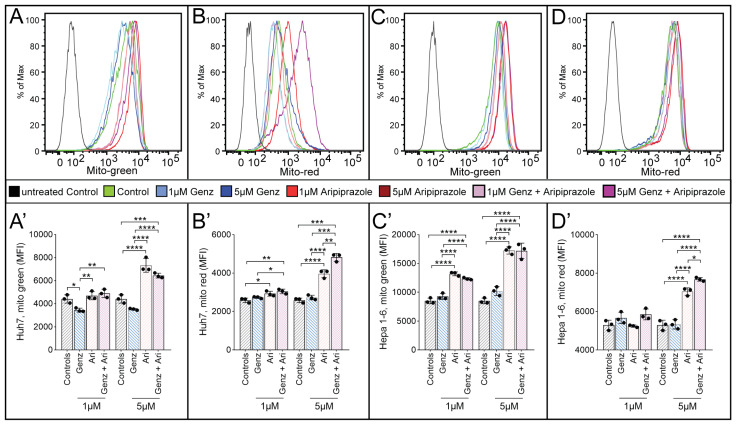
Aripiprazole enhances mitotracker incorporation into cancer cells. Determination of mitotracker green medium fluorescence intensity (MFI) ((**A**,**A’**), Huh7 cells and (**C**,**C’**), Hepa 1-6 cells) as well as mitotracker red MFI ((**B**,**B’**), Huh7 cells and (**D**,**D’**), Hepa 1-6 cells) by FACS analysis. Huh7 cells (**A**,**A’**,**B**,**B’**) and Hepa 1-6 cells (**C**,**C’**,**D**,**D’**) were treated with 1 µM/5 µM Genz or 1 µM/5 µM aripiprazole (Ari) and combinations of both drugs for five days. Mitotracker reagents were added as described in the methods section for 30 min, and cells were consequently harvested and analyzed by flow cytometry. Shown is one representative experiment out of two; n = 3 for each condition; note: identical controls were plotted twice, one for each condition (**A’**–**D’**). Bar diagrams show mean values ± SD. Significances calculated with an one-way ANOVA test are: *, *p* ≤ 0.05; **, *p* < 0.01; ***, *p* < 0.001; ****, *p* < 0.0001.

To verify whether not only lysosomal, but also mitochondrial functions were affected by treatment with Genz and aripiprazole, Huh7 and Hepa 1-6 cells were stained with mitotracker reagents. Mean fluorescence intensity (MFI) for mitotracker green, an indicator for mitochondrial mass, appeared largely unchanged upon 1 µM aripiprazole treatment of Huh7 cells, while treatment with the same agent at 5 µM resulted in a marked increase (Figure 6A,A’). Application of 1 µM or 5 µM Genz in Huh7 cells resulted in a reduction in fluorescence intensity compared to untreated controls (Figure 6A,A’). In contrast, mitotracker green intensity increased significantly after application of 1 µM or 5 µM of aripiprazole to Hepa 1-6 cells (Figure 6C,C’, quantification). To also investigate changes in mitochondrial membrane potential, mitotracker red was applied. This staining was most intense in both cell lines upon treatment with 5 µM aripiprazole or in a combination of 5 µM aripiprazole together with 5 µM Genz (Figure 6B,B’, Huh7; Figure 6D,D’, Hepa 1-6).

Despite the observed changes in mitochondrial mass as detected by mitotracker green staining, expression of several mitochondrial proteins such as HSP60, PDH, PHB1, SDHA, and VDAC did not show robust differences in response to drug treatment, with levels varying between both cell lines (Supplementary Figure S3A,C–G, quantification). In addition, the ER-marker proteins PERK and Ire1α appeared almost unchanged upon Genz or aripiprazole treatment of Huh7 and Hepa 1-6 cells (Supplementary Figure S3B,H,I, quantification), suggesting that these drugs may not have major effects on ER-function.

### 2.7. Genz and Aripiprazole Treatment Synergistically Lower Mitochondrial Respiration and Glycolysis

**Figure 7:**
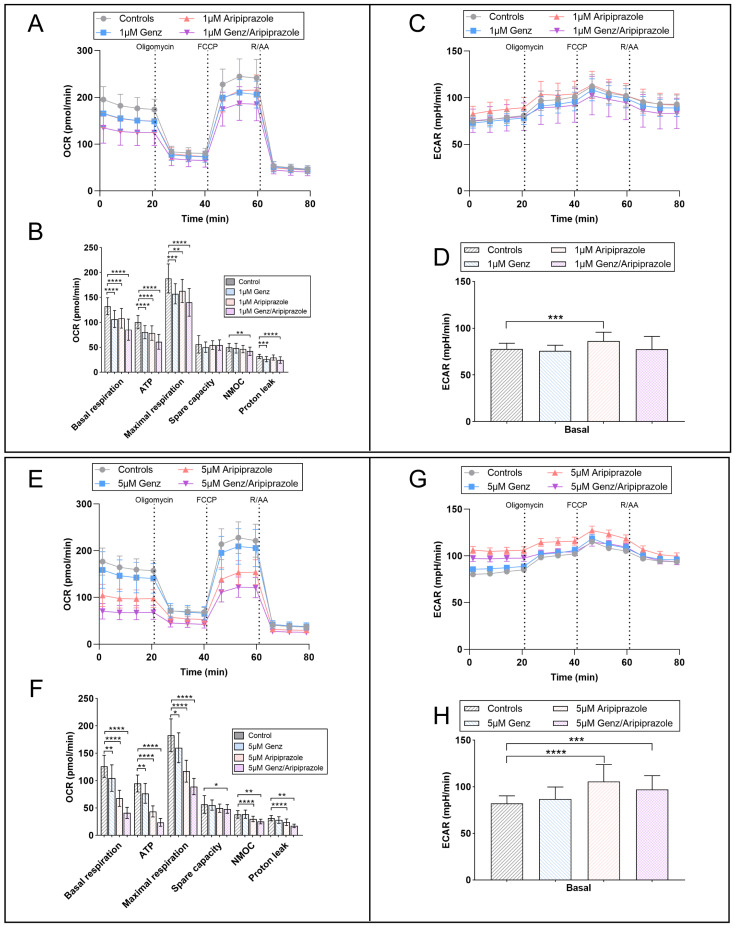
Seahorse mito-stress analysis showing differences in OCR and the ECAR profiles of cells treated with different Genz and aripiprazole concentrations. (**A**) Mitochondrial activity of Hepa 1-6 cells cultured in the presence of 1 µM Genz, 1 µM aripiprazole, or a combination of both drugs for 5 days was analyzed using the mitochondrial stress test (Seahorse). (**B**) Basal respiration, ATP-linked respiration, and maximal respiration were calculated from the flux profile. Significance was calculated using a one-way ANOVA test (n = 20 to 24), *, *p* ≤ 0.05; **, *p* < 0.01; ***, *p* < 0.001; ****, *p* < 0.0001; (OCR, oxygen consumption rate; NMOC, non-mitochondrial oxygen consumption). (**C**) Extracellular acidification rate (ECAR) of the same cells shown in (**A**). (**D**) Basal ECAR is displayed as an estimation of the compensatory glycolytic activity of the cells in response to drug treatment. (**E**) OCR and (**F**) calculated respiratory parameters of cells treated with 5 µM Genz, 5 µM aripiprazole, or 5 µM Genz/5 µM aripiprazole, respectively. (**G**) ECAR of cells cultured with 5 µM of the drugs and (**H**) corresponding basal ECAR; n = 20 to 24 for each treatment condition. Graphs and bar diagrams show mean values ± SD.

In order to verify the results obtained from the mitotracker experiments, we performed a Seahorse metabolic flux assay to obtain deeper insight into the effect of aripiprazole or Genz treatment on mitochondrial respiration by monitoring oxygen consumption rates (OCR). Based on this analysis, we observed a significant reduction in basal respiration, ATP-dependent respiration, and maximal respiration in Hepa 1-6 cells treated with 1 µM Genz or aripiprazole, either alone or in combination, for five days (Figure 7A,B). Treatment with the compounds using the higher concentration of 5 µM resulted in a stronger reduction in these parameters (Figure 7E,F). The impact of aripiprazole was more pronounced than the effect of Genz, and the strongest impact on mitochondrial respiration was observed in combinations of both drugs. In contrast, only treatment with aripiprazole, either alone or in combination with Genz, caused a small increase in the extracellular acidification rate (ECAR), suggesting that cells only show a limited ability to compensate for the inhibition of mitochondrial ATP production by upregulating glycolysis (Figure 7C,D,G,H).

### 2.8. Aripiprazole, Particularly in Combination with Genz, Inhibits Tumor Cell Growth and Enhances the Efficacy of the Cytostatic Drugs Sorafenib and Doxorubicin

**Figure 8:**
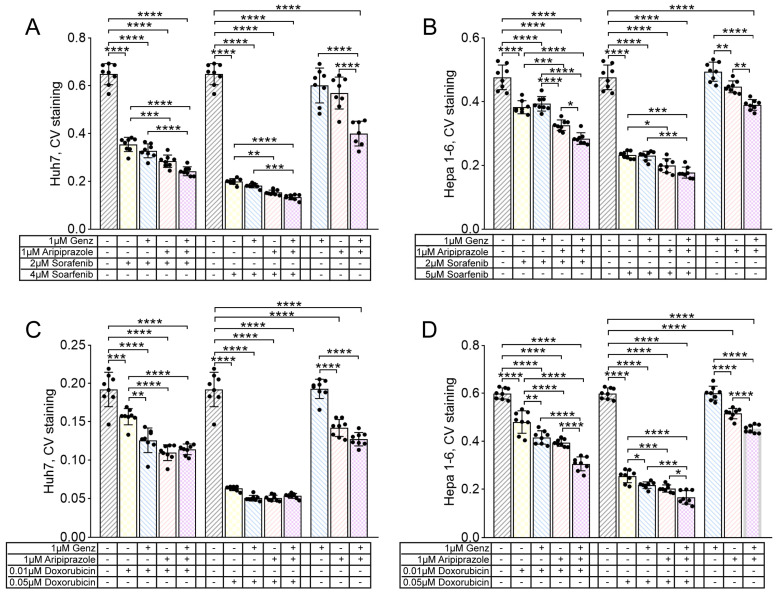
Hepatocellular tumor cell growth is impaired by the administration of aripiprazole in combination with Genz and the cytostatic drugs sorafenib and doxorubicin. (**A**–**D**) Huh7 and Hepa 1-6 cells were treated with 1 µM Genz, 1 µM aripiprazole, or 1 µM Genz/1 µM aripiprazole, in combination with either sorafenib or doxorubicin as described in the method section; note: controls were plotted twice on each diagram. (**A**,**C**) crystal violet proliferation assay of Huh7 cells treated for 48 h with combinations of Genz/aripiprazole/sorafenib (**A**) or Genz/aripiprazole/doxorubicin (**C**). (**B**,**D**) crystal violet proliferation assay of Hepa 1-6 cells treated for 48 h with combinations of Genz/aripiprazole/sorafenib (**B**) or Genz/aripiprazole/doxorubicin (**D**); n = 8 for each treatment condition; note: identical controls were plotted twice (**A**–**D**), one for each low and high sorafenib and doxorubicin concentration. Bar diagrams show mean values ± SD. Significances calculated with an one-way ANOVA test are: *, *p* ≤ 0.05; **, *p* < 0.01; ***, *p* < 0.001; ****, *p* < 0.0001.

In order to investigate potential combinatorial effects of the two compounds with standard cytostatic agents used to treat HCC, Genz and aripiprazole were added to Huh7 and Hepa 1-6 cells at a concentration of 1 µM for 48 h, and cell viability was determined by crystal violet assay. Genz application did not lead to a marked reduction in crystal violet staining intensity in Huh7 or Hepa 1-6 cells (Figure 8A–D). However, application of aripiprazole, either alone or in combination with Genz, led to moderate reduction in cell viability in both cell lines (Figure 8A–D).

Treatment of cells with the multi-tyrosine kinase inhibitor sorafenib or the chemotherapeutic agent doxorubicin together with Genz and/or aripiprazole resulted in a stronger inhibition of cell viability compared to single treatment. This was particularly apparent for aripiprazole or the combination of Genz/aripiprazole applied together with either 2 µM or 4 µM of sorafenib (Figure 8A). Similarly, the combination of Genz and aripiprazole applied together with 0.01 µM or 0.05 µM of doxorubicin also led to a stronger reduction in cell viability of Huh7 cells compared to doxorubicin monotherapy (Figure 8C). Similarly, the growth of Hepa 1-6 cells treated with 2 µM or 5 µM sorafenib (Figure 8B) or 0.01 µM or 0.05 µM doxorubicin (Figure 8D) was effectively reduced by co-administration of aripiprazole or aripiprazole/Genz.

### 2.9. Genz and Aripiprazole in Combination with Sorafenib or Doxorubicin Impair Growth of Hepatoma Tumor Spheroids

**Figure 9:**
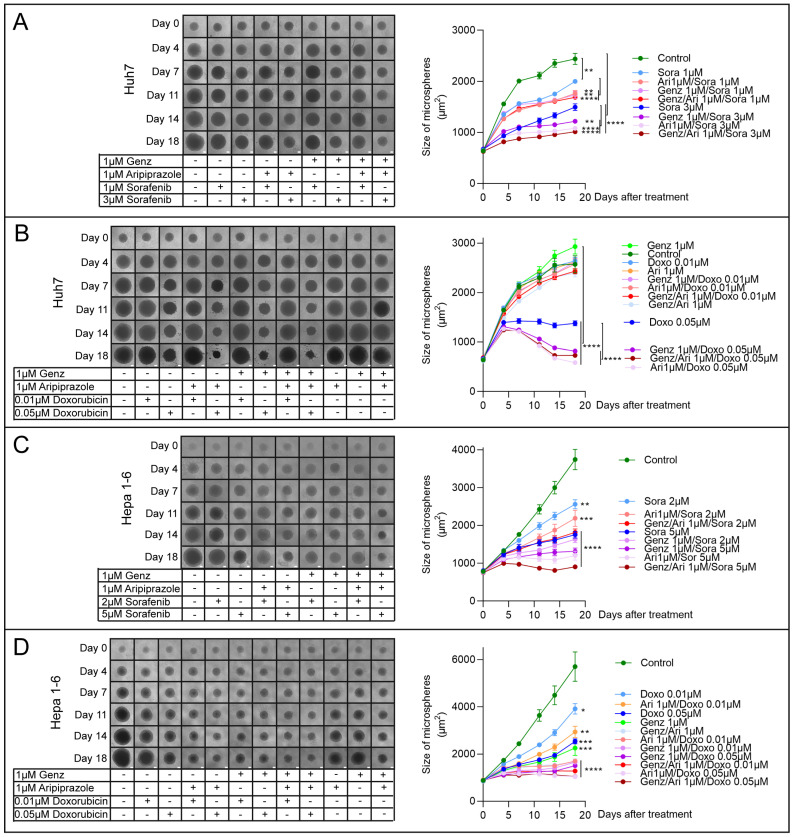
Genz and/or aripiprazole combinations with sorafenib or doxorubicin impair growth of tumor spheroids. (**A**–**D**) 10^3^ Huh7- and Hepa 1-6 cells were seeded in 96-well ultra-low attachment plates. HCC tumor microspheres formed within 24 h (Supplementary Figure S4). Common tissue culture medium was replaced as described in the method section with 1 µM Genz, 1 µM aripiprazole (Ari), or 1 µM Genz/1 µM aripiprazole in combination with either sorafenib or doxorubicin as indicated in the figure on day three. Medium, including the drugs, was exchanged twice a week, and the size of the tumor microspheres was determined by ImageJ, version 1.54d. (**A**,**B**) Huh7 cells were treated for 18 d with combinations of Genz/aripiprazole/sorafenib (**A**) or Genz/aripiprazole/doxorubicin (**B**). (**C**,**D**) Hepa 1-6 cells were treated for 18 d with combinations of Genz/aripiprazole/sorafenib (**C**) or Genz/aripiprazole/doxorubicin (**D**); n = 8 for each treatment condition. Significances calculated with an one-tailed *t*-test (controls vs. treated, or as indicated at day 18) are: *, *p* ≤ 0.05; **, *p* < 0.01; ***, *p* < 0.001; ****, *p* < 0.0001.

The inhibitory potential of Genz and aripiprazole co-administered with sorafenib or doxorubicin was further tested in a tumor microsphere model. This three-dimensional cell system allows us to investigate the effectiveness of drug combinations over the extended time period of 18 days. This analysis showed that the growth of Huh7 microspheres was almost completely blunted by the administration of 3 µM sorafenib together with either 1 µM Genz or 1 µM aripiprazole (Figure 9A). The most effective treatment combination consisted of Genz/aripiprazole, each at 1 µM concentration, together with 3 µM sorafenib. Similarly, Huh7 spheroids treated with 1 µM Genz or 1 µM aripiprazole together with 0.05 µM doxorubicin displayed a substantial reduction in tumor spheroid size (Figure 9B). The strongest growth reduction was achieved using a combination of 1 µM Genz/1 µM aripiprazole together with 0.05 µM doxorubicin.

Administration of 5 µM sorafenib combined with 1 µM Genz/aripiprazole to Hepa 1-6 tumor microspheres most effectively impaired spheroid growth, followed by aripiprazole/sorafenib and Genz/sorafenib (Figure 9C). A combination of 0.05 µM doxorubicin with 1 µM Genz/aripiprazole led to a complete stop of tumor microsphere growth (Figure 9D). Furthermore, the combination of Genz/doxorubicin or aripiprazole/doxorubicin also resulted in significant growth inhibition (Figure 9D). Taken together, these experiments showed that administration of aripiprazole or Genz, either individually or more effectively in combination, significantly enhanced the effect of the multi-tyrosine kinase inhibitor sorafenib or the chemotherapeutic agent doxorubicin on HCC cell growth inhibition.

## 3. Discussion

Hepatocellular carcinoma (HCC) is one of the leading causes of cancer deaths worldwide [1]. Due to late detection, approximately 40% of HCC patients often present with advanced disease. Treatment options are primarily restricted to supportive care and systemic therapies with multi-tyrosine kinase inhibitors such as sorafenib [42,43,44]. More recently, the checkpoint inhibitors pembrolizumab and nivolumab in combination with the angiogenesis inhibitor bevacizumab have been approved for the treatment of HCC [45].

Here, we demonstrate the impact of the GCS inhibitor Genz and the CAD and anti-psychotic drug aripiprazole, together with sorafenib and the DNA-intercalating agent doxorubicin [46], on the growth of Huh7 and Hepa 1-6 hepatocellular cancer cells and cancer spheroids. We show that both Genz and aripiprazole interfered with cancer cell proliferation by reducing the proportion of cells entering the S-phase of the cell cycle. This was observed already at low concentrations but was more pronounced at higher concentrations. Only a small proportion of cells treated with a combination of 5 µM Genz and aripiprazole were able to enter S-phase, with many cells showing evidence of a G2 cell cycle arrest. This observation went along with reduced expression of the cyclins A and E. Cyclin A is upregulated during S-phase and expressed at high levels during G2, as it stimulates the transition of cells to mitosis [47]. Cyclin E expression is mostly restricted to late G1 and S-phase [48]. High expression of Cyclins A and E has been documented in various human cancer types [47,48]. A reduction in those cyclins due to Genz/aripiprazole treatment suggests that these compounds impact cancer cell growth. At concentrations of 5 µM, particularly a combination of Genz and aripiprazole, induced a small but significant increase in the number of apoptotic and necrotic cancer cells as compared to untreated controls, which might be of advantage. However, it is possible that high concentrations of these compounds may also affect the viability of non-tumorous cells, causing dose-limiting toxicity. It has been shown that high doses of cationic amphiphilic drugs cause phospholipidosis with lipid accumulation [49]. Therefore, careful titration is essential.

So far, the GCS-inhibitor used in this study (Genz-123346) has not been applied to patients. A similar compound, eliglustat (Genz-112638), is approved for the treatment of Gaucher disease and evaluated in some clinical studies for childhood cancers [17,50]. Eliglustat differs from Genz-123346 by a single additional CH_2_-group in the amphiphilic chain. Single-dose administration of 30 mg eliglustat/kg to healthy volunteers led to serum concentrations of approximately 1 µM after 12 h [51], one of the concentrations that we have chosen in our experiments.

Additionally to GCS-inhibition with Genz, we used aripiprazole in our study. Several CADs, including aripiprazole, have already been described to reduce cancer cell growth in two-dimensional cell culture models [33]. We have chosen the antipsychotic antidepressant drug aripiprazole since it is widely prescribed and well tolerated. The effect of this drug as an antidepressant may be of advantage for its re-purposing as an anti-cancer agent, as the psychological impact of cancer diagnosis, which can cause deep depression, may not be underestimated. The concentration of 1 µM of aripiprazole used in the tissue culture experiments of our study is almost equivalent to the concentrations in serum of patients 24 h after application of a single 30 mg dose of the drug [52].

The effect of Genz and aripiprazole, either alone or in combination therapy, on cancer cell viability in two-dimensional cell culture or tumor spheroids was significant but rather modest. However, both drugs increased the growth inhibitory effect of the multi-tyrosine kinase inhibitor sorafenib or the chemotherapeutic agent doxorubicin in both monolayer and spheroid culture. HCC patients frequently develop drug resistance [53]. Interestingly, sorafenib treatment has been associated with elevated GCS expression [54], which itself was reported to enhance drug resistance [55]. Inhibition of glucosylceramide synthase with specific GCS-inhibitors may therefore represent an effective approach in overcoming the resistance of HCC cells to sorafenib treatment.

Concentrations of 1 µM aripiprazole and Genz and more effective combinations of both drugs sufficed to impair mitochondrial respiration. This may represent the main mechanism by which these compounds reduce cancer cell growth.

In the present study, we have explored the role of GCS-inhibition with Genz-123346 together with the cationic amphiphilic drug aripiprazole in combination therapy with the multi-tyrosine kinase inhibitor sorafenib and the chemotherapeutic drug doxorubicin on the growth of hepatocellular cancer cells in monolayer and tumor spheroid culture. Our results show that combinations of these drugs could represent a promising approach to treat patients suffering from HCC and warrant further testing in a pre-clinical in vivo-model.

## 4. Materials and Methods

### 4.1. Chemicals and Antibodies

Genz, (Genz-123346), (*N*-[(1*R*,2*R*)-1-(2,3-dihydro-1,4-benzodioxin-6-yl)-1-hydroxy-3-pyrrolidin-1-ylpropan-2-yl]nonanamide) (Chess, Mannheim, Germany); aripiprazole (Thermo, Sindelfingen, Germany, #457990010); doxorubicin (VWR, Darmstadt, Germany #CAYM15007-5); sorafenib (LC Laboratories, Wobum, MA, USA, #S-8599).

Primary antibodies used: cyclin A (Santa Cruz, Heidelberg, Germany, sc-596); cyclin E (Santa Cruz, sc-481); pyruvat dehydrogenase (PDH, Cell Signaling, Danvers, MA, USA, CS-3205); HSP60 (Cell Signaling, CS-4870); SDHA (Cell Signaling, CS-5839); PHB1 (Cell Signaling, CS-2426); VDAC (Cell Signaling, CS-4661); PERK (Cell Signaling, CS-3192); IRE1a (Cell Signaling, CS-3294); and Lc3 (Sigma, Munich, Germany, #L8918), all raised in rabbit, 1:1000 diluted in 5% BSA in TBS/0.1% tween-20.

GAPDH (Santa Cruz, sc-25778), 1:2000, or β-Tubulin (Cell Signaling, CS-2128), 1:3000, in 5% BSA in TBS/0.1% tween-20 were chosen as loading controls. As secondary antibodies, anti-rabbit HRP (Dako, Hamburg, Germany, #P0399), 1:1000 in 5% BSA in TBS/0.1% tween-20 were used.

### 4.2. Cell Culture

Human (Huh7 and HepG2) as well as mouse (Hepa 1-6) hepatocellular carcinoma cells (kindly provided by U. Klingmüller from our institution) were cultivated in DMEM, a high-glucose medium (Sigma, #D5796), supplemented with 1% penicillin/streptomycin (Invitrogen, Waltham, MA, USA, #15140-122), 1% hepes (Invitrogen, #15630-056), and 10% FCS (Invitrogen, #A5256801). Medium was changed two to three times per week, and cells split at ~90% confluency.

### 4.3. 5-Bromo-2′-Desoxyuridine (BrdU) Incorporation

Cells were cultivated in 10 cm dishes under the presence/absence of 1 µM and 5 µM Genz or aripiprazole or combinations of both for 4 days. Cells were counted, and 10^5^ cells were split in triplicates into 6-well plates. Treatment continued overnight. BrdU (Sigma, #B5002) was applied to a final concentration of 10 µM. Cells were further cultivated for 4 h, consecutively washed with PBS, trypsinized, and centrifuged (500× *g*, 5 min). After washing with PBS, cells were suspended in 200 µL of ice-cold PBS by pipetting up and down. Cells were then fixed by dropwise addition of 800 µL of ice-cold ethanol while gently vortexing the mixture. After overnight fixation at −20 °C, cells were counted and equal numbers were transferred to FACS tubes pre-filled with 3 mL of PBS/1% BSA, centrifuged as before, and the supernatant decanted. Cells were suspended in 1 mL of 2 M HCl/0.5% Triton X-100 and incubated for 30 min at room temperature. The cell mixture was shortly and gently vortexed every 10 min. After centrifugation and discarding the supernatant, cells were suspended in 1 mL of 0.1 M Na_2_B_4_O_7_, pH 8.5, and shortly (~5 min) incubated on ice. A total of 3 mL of cold PBS/1% BSA were added, the cells were centrifuged, the supernatant was decanted, and the cells were then stained with 100 µL of a 1:20 diluted anti-BrdU antibody (FITC-anti-BrdU, Biolegend, San Diego, CA, USA, #364104) for 30 min at room temperature in the dark. A total of 1 mL PBS/1% BSA was added, the cells centrifuged as before, and the supernatant carefully discarded. Cells were resuspended in 200 µL of 38 mM sodium citrate, 54 µM propidium iodide, and 24 µg/mL RNAse A (AppliChem, Darmstadt, Germany, #A3832,0250) and incubated for 30 min at 37 °C in the dark. Cells were subsequently measured by FACS (Canto II) with Diva software, version v9.0. Quantification was performed using FlowJo software, version 3.0.

### 4.4. Cell Viability Assay

Huh7 and Hepa 1-6 cells were cultivated in the presence of 1 µM/5 µM Genz or aripiprazole for six days as described above. The cells were harvested by trypsination, washed with PBS, and suspended to 10^5^ cells in 100 µL of annexin binding buffer from the FITC Annexin V Apoptosis Detection Kit with 7-AAD (Biolegend, #640922). After transferring 100 µL of the cell suspension into FACS tubes, 5 µL of annexin V and 7AAD viability staining solution from the kit were added, and cells were incubated for 15 min at room temperature in the dark. Then 400 μL of Annexin V Binding Buffer were added to each tube, and the cells were analyzed by FACS Canto as described.

### 4.5. Lyso- and Mito-Tracker Assays

In order to gain a crude overview of lysosomal and mitochondrial function, Huh7 and Hepa 1-6 cells were cultivated in the presence of Genz or aripiprazole as described above. Cells were harvested on day six after treatment and subsequently stained with lysotracker green (CS-8783), 1:20,000 diluted, and mitotracker green (Biotium, Freemont, CA, USA, MitoView green, 70054) or red (CS-9082), each 1:10,000 diluted, according to the manufacturer’s instructions. Cells were washed and analyzed by FACS analysis.

### 4.6. Electron Microscopy of Genz and Aripiprazole Treated Hepa 1-6 Cells

Hepa 1-6 cells cultured in low-adhesion culture dishes (Greiner, Gremsmünster, Austria, #632180) were treated with either 5 μM Genz or aripiprazole for 6 days with one medium change after three days. Cells were harvested by centrifugation (5 min/1000 rpm), fixed in 2% formic aldehyde/2% glutaric aldehyde/1 mM CaCl_2_/1 mM MgCl_2_/100 mM cacodylate buffer pH 7.2, following post-fixation in 1% OsO_4_, Uranyl en bloc staining (1% uranyl-acetate in 75% ethanol), dehydration in ethanol, and Epon-embedding (Serva, Heidelberg, Germany). Ultrathin sections were cut at 50 nm nominal thickness (UCT, Leica, Wetzlar, Germany) and contrasted with uranyl and lead. Images were taken with an EM910 (Carl Zeiss Oberkochen, Germany) equipped with a CCD-Camera (TRS, Morenweis, Germany).

### 4.7. Western Blotting of Genz-Treated Cells

Huh7 and Hepa 1-6 cells were treated with 1 µM and 5 µM of Genz or aripiprazole for six days as described. At ~70% confluency, cells were washed with PBS and lysed in digitonin lysis buffer [20 mM HEPES-NaOH buffer (pH 7.4) containing 25 mM KCl, 250 mM sucrose, 2 mM MgCl_2_, with freshly added 1% digitonin (Serva), complete protease inhibitors (Roche, Ludwigsburg, Germany), phospho-stop (Roche), and 0.5 mM DTT] by vigorously pipetting up and down. The lysates were incubated on ice for 30 min and then centrifuged at 13,000 rpm at 4 °C for 5 min. Supernatants were collected and protein content determined using Bradford reagent (Sigma, #B6916). A total of 20 µg of protein was applied to either a 10% or 12.5% SDS gel, depending on the molecular weight of the protein. The proteins were blotted on a nitrocellulose membrane (GE-Healthcare, Amersham, UK), and unspecific binding was blocked with 5% BSA in TBS/0.1% tween-20. The membranes were then incubated with primary antibodies at 4 °C overnight. The blots were washed three times with TBS/0.1% tween 20 and incubated with secondary AB at room temperature for 1 h. After three times washing as above, bands were visualized with ECL solution (Biorad, Hercules, CA, USA, #170-5060) in a ChemiDoc TM Imaging System (Biorad). Quantification was performed with Image J.

### 4.8. Sphingolipid Extraction of Hepatocellular Carcinoma Cells

Hepatocellular cancer cells were cultured as triplicates in 10 cm cell culture dishes in the presence of 1 µM and 5 µM Genz, 1 µM and 5 µM aripiprazole, or combinations of both for four days. The medium was exchanged, and treatment continued for one or two more days until a confluency of ~80 to 90% was reached. Cells were washed two times with PBS and harvested with a cell scraper. The pellets were extracted with 1 mL CHCl_3_/CH_3_OH/H_2_O 10:10:1 by vol. in an ultrasound water bath at 37 °C for 15 min. Supernatants were collected after centrifugation (10 min, 3000 rpm), and the pellet was extracted a second time with 1 mL CHCl_3_/CH_3_OH/H_2_O 10:10:1 by vol. and a third time with 1 mL CHCl_3_/CH_3_OH/H_2_O 30:60:8 by vol. as before. Combined supernatants were evaporated by a stream of nitrogen. The cell pellet was air dried and solved overnight in 1 M aqueous NaOH followed by a BCA protein determination with reagents A and B (Pierce, Waltham, MA, USA, A, #23223; B, #1859078). To an aliquot of 12.5 µg of the respective amount of protein, an internal standard containing sphingomyelin, PC, ceramide, and hexosylceramide was added and measured by LC-MS/MS as described elsewhere [9,56].

For TLC analysis of phospholipids and sphingomyelin, an aliquot of the lipid crude extracts corresponding to 25 µg of protein was loaded on HPTLC plates (Merck, Darmstadt, Germany). The plate was developed in running solvent chloroform/methanol/water 65:25:4. Lipids were visualized with copper spray reagent consisting of 10% CuSO_4_ in 8% H_3_PO_4_ at 180 °C for ~10 min.

### 4.9. Seahorse Mito-Stress Assay

The Agilent Seahorse XF cell mito-stress test evaluates the mitochondrial function by directly measuring the oxygen consumption rate (OCR) and the extracellular acidification rate (ECAR) of live cells on the Seahorse XFe96 flux analyzer (©Agilent Technologies Inc., Wilmington, NC, USA). In order to test whether drug treatment would alter mitochondrial respiration, Hepa 1-6 cells were incubated either with 1 µM or 5 µM Genz or aripiprazole or combinations of both drugs under 5% CO_2_ at 37 °C for four days. Cells were split and seeded in drug-supplemented medium at a density of 25,000 cells for each condition except for 5 µM Genz/aripiprazole, from which 30,000 cells per well were added into a 96-well seahorse plate. Cells were allowed to adhere overnight. The cell mito-stress test was performed as described in the manufacturer’s protocol (Kit 103015–100) using the following reagent’s final concentrations; oligomycin: 2 µM, FCCP: 3.5 µM, and rotenone/antimycin A: 0.5 µM each. Cells reached ~95% confluency under all treatment conditions as verified with an Incucyte live cell imaging and analysis system (Sartorius, Göttingen, Germany) and were additionally normalized at the end of the measurement to a crystal violet staining.

The sequential exposure of cells to oligomycin, carbonyl cyanide-4 (trifluoromethoxy) phenylhydrazone (FCCP), and rotenone/antimycin, all inhibitors of different mitochondrial electron chain complexes, allows the analysis of the changes in mitochondrial respiration under different culturing conditions or treatments. Oligomycin inhibits ATP synthase (complex V) and is injected first in the assay following basal measurements. It decreases electron flow through the electron transfer chain (ETC), resulting in a reduction in mitochondrial respiration. This decrease in OCR is linked to cellular ATP production. FCCP is an uncoupling agent that collapses the proton gradient and disrupts the mitochondrial membrane potential. As a result, electron flow through the ETC is uninhibited, and oxygen consumption by complex IV reaches the maximum. The third injection is a mixture of rotenone, a complex I inhibitor, and antimycin A, a complex III inhibitor. This combination shuts down mitochondrial respiration and enables the calculation of non-mitochondrial respiration driven by processes outside the mitochondria.

### 4.10. Cell Proliferation Assay Using Crystal Violet

Huh7 and Hepa 1-6 cells were seeded at 4 × 10^3^ and 2 × 10^3^ cells, respectively, into 96-well cell culture plates. Eight wells were used as repeats for each treatment condition. Different concentrations of doxorubicin and sorafenib were pre-tested in dose escalation determination experiments. Doses of doxorubicin and sorafenib, leading to approximately 20% and 50% reduction in cell proliferation within 48 h, were used together with aripiprazole and/or Genz. The concentration of aripiprazole and Genz has been adjusted in cell culture medium to the serum concentration reached in patients treated with the drugs, which reached levels of ~1 µM within 12 h to 24 h. Cells were allowed to adhere overnight prior to treatment. Plates were then treated with Genz and aripiprazole in combinations with the chemotherapeutics sorafenib or doxorubicin as indicated in the respective figures in hepatocyte medium for 48 h. Cells were fixed using 4% PFA in PBS for 10 min, washed with PBS, and air-dried for ~30 min. Afterwards, 100 µL of the crystal violet solution (0.1% (*w*/*v*) crystal violet in 20% ethanol) was applied to each of the wells, and the cells were incubated for 30 min. The plate was rinsed four times with dH_2_O and air-dried overnight. The remaining crystal violet, adhering to the cells, was resolved in 100 µL of 10% aqueous acetic acid while the plates were shaken at 300 rpm for 30 min. Color was measured at 585 nm using a Synergy H1 microplate reader (BioTek Instruments GmbH, Bad Friedrichshall, Germany).

### 4.11. Tumor Spheroids

To initiate spheroid formation 1000 cells in 200 µL of regular growth medium were pipetted into each well of a 96-well round-bottom ultralow attachment plate (Corning, Kaiserslautern, Germany, CORN7007) followed by centrifugation at 450 × *g* for 10 min. Spheroids were allowed to form for three days (Supplementary Figure S4). Then half of the medium was carefully removed and wells refilled by addition of the same volume of double-concentrated Genz, aripiprazole, sorafenib, and doxorubicin combinations as indicated in Figure 9. Spheroid growth was monitored twice a week using a microscope (Nikon, Minato, Japan, Eclipse TS2) for a total of three weeks, and the medium, including the respective drugs, was partly exchanged on the same day. Spheroid size was determined with ImageJ.

## Data Availability

All data are available within the main body of the manuscript or in the Supporting Information.

## Supplementary Materials

The supporting information can be downloaded at: https://www.mdpi.com/article/10.3390/ijms26010304/s1.
