# Supplementary material for: Glucosylceramide Synthase Inhibition in Combination with Aripiprazole Sensitizes Hepatocellular Cancer Cells to Sorafenib and Doxorubicin
Source: Int J Mol Sci. 2024 Dec 31;26(1):304. doi: 10.3390/ijms26010304 (PMC11720485; doi:10.3390/ijms26010304)
Supplement: Supplementary file 1 [file ijms-26-00304-s001.zip › ijms-3356951-supplementary.pdf]

Glucosylceramide synthase-inhibition in combination with aripiprazole sensitizes hepatocellular cancer cells to sorafenib- and doxorubicin.

Richard Jennemann, Martina Volz, Roberto Carlos Frias-Soler, Almut Schulze, Karsten Richte[4], Sylvia Kaden, and Roger Sandhoff

Supporting Figures:

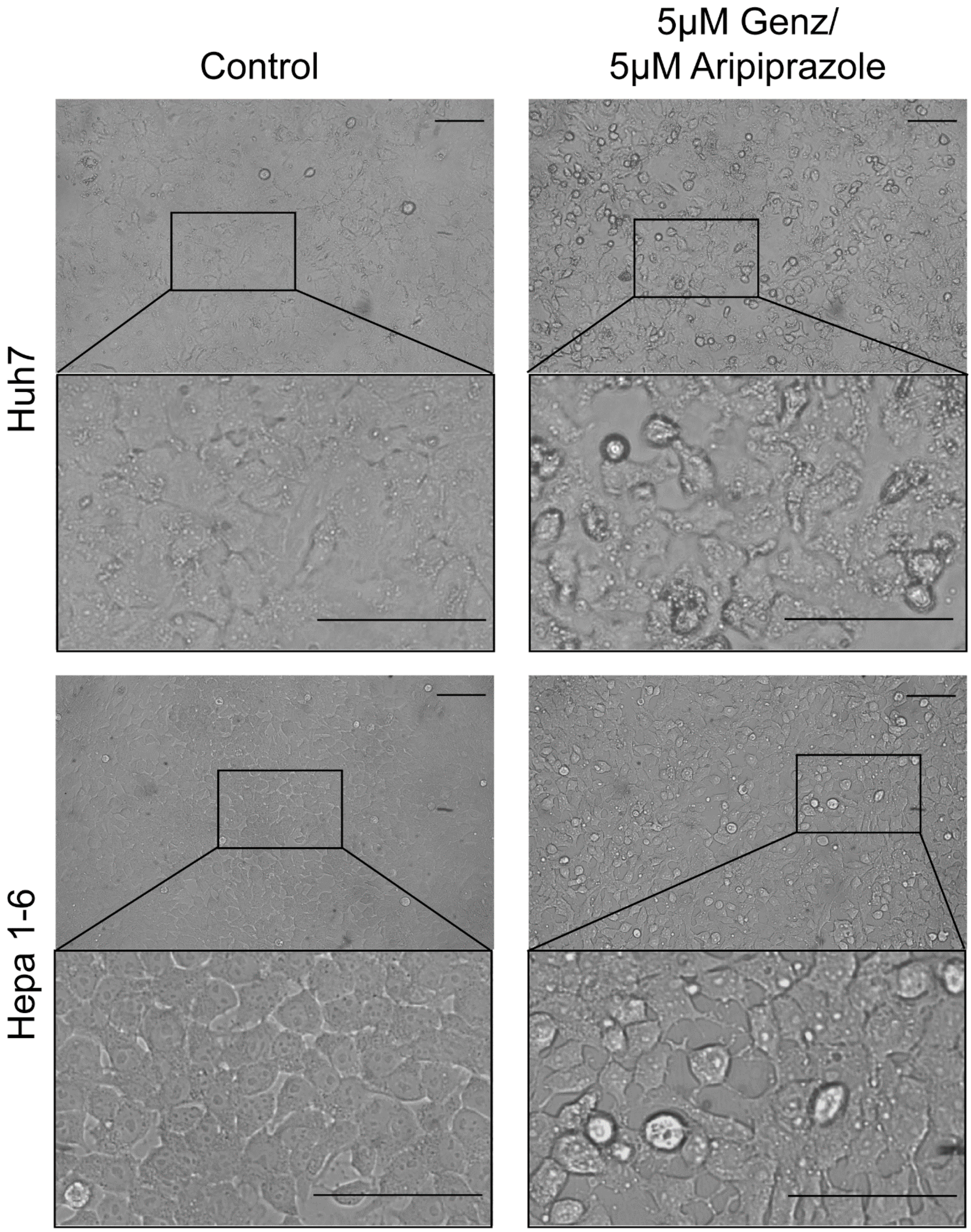

Supporting Figure S1
Cancer cells arrest upon aripiprazole/Genz treatment in G2/M phase of the cell cycle. Treatment of Huh7 and Hepa 1-6 hepatocellular cancer cells with 5µM aripiprazole or 5µM Genz caused an arrest in G2/M phase indicated by significant elevation of rounded cells corroborating the results from the cell cycle analysis shown in Figure 1. In addition, both cell lines treated with the drugs showed an increased number of lysosomal structures within the cytosol (white dots within the cytosol).

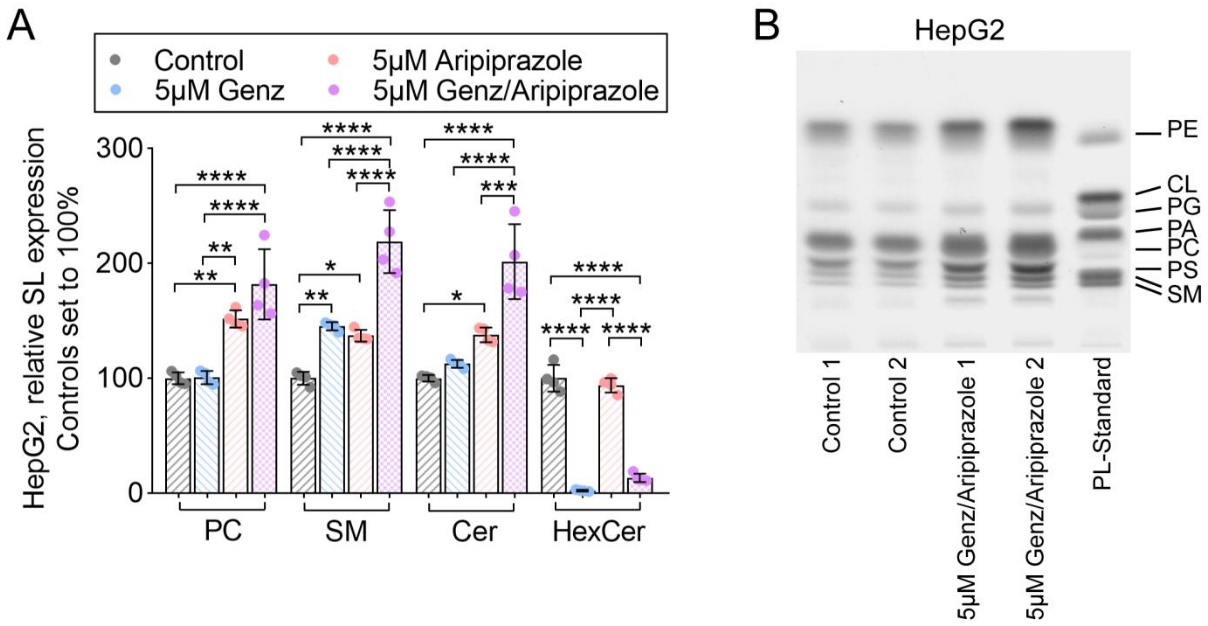

Supporting Figure S2

Sphingolipid and phospholipid synthesis increases in HepG2 hepatocellular cancer cells upon treatment with aripiprazole or Genz. (A) Treatment of HepG2 hepatoma cells with 5µM aripiprazole or/and Genz caused an increase of phosphatidylcholine, sphingomyelin and ceramides similar as shown for Huh7 and Hepa 1-6 cells; n=3 for each condition, mean values ± SD. Significances calculated by an one-way Anova-test are: *, $p \leq 0.05$; **, $p < 0.01$; ***, $p < 0.001$; ****, $p<0.0001$. TLC analysis reflected a general increase of phospholipids in HepG2 cells treated with 5µM Genz/5µM aripiprazole (B).

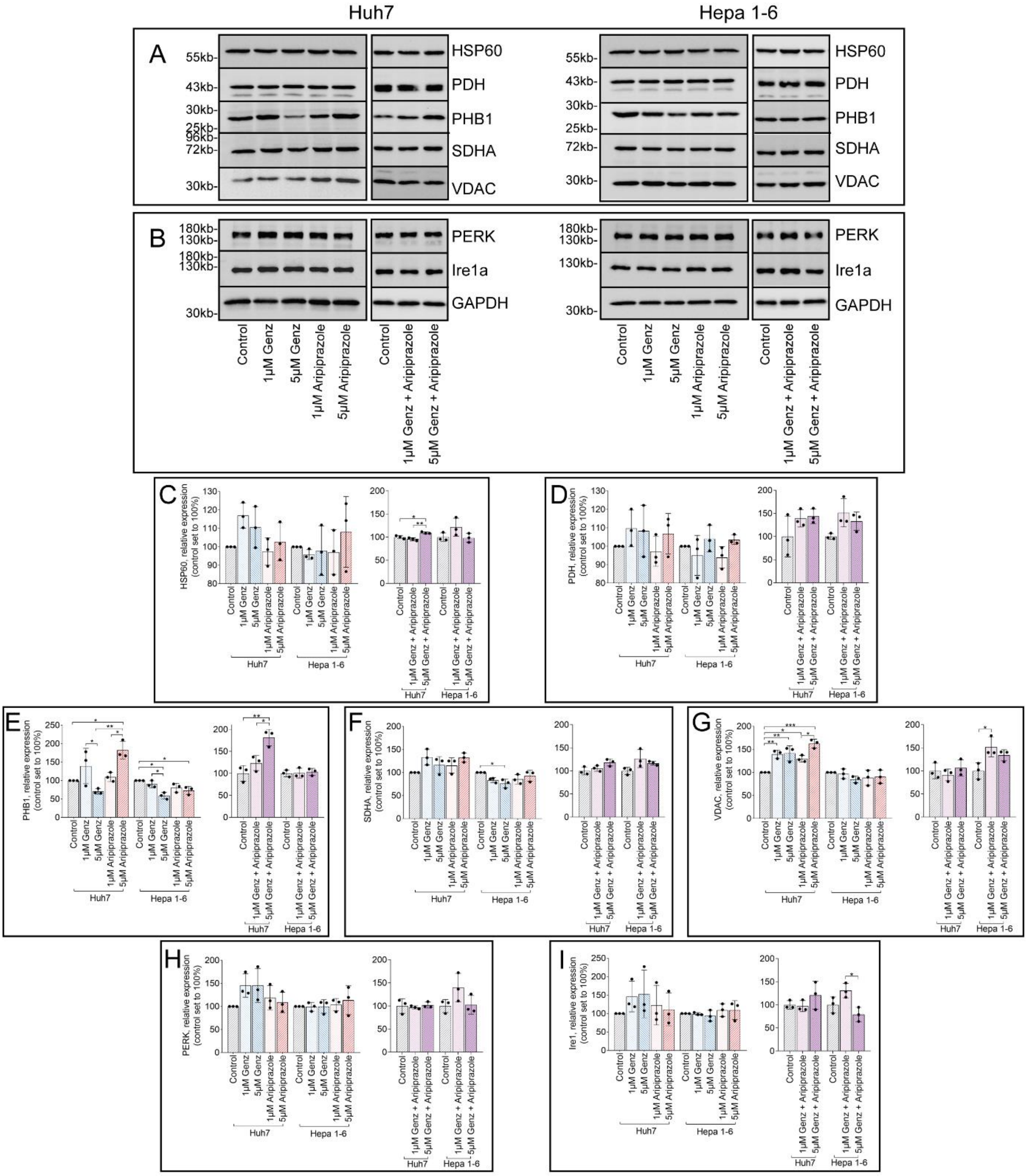

Supporting Figure S3

Western blot analysis of protein extracts from Huh7 and Hepa 1-6 hepatoma cells. Clear differences in the expression of proteins involved in mitochondrial function ((A) blot, C to G, quantification) such as heat shock protein 60 (HSP60 (C)), pyruvate dehydrogenase (PDH, (D)), prohibitin-1 (PHB1 (E)), Succinate dehydrogenase complex, subunit A (SDHA (F)), voltage-dependent anion-selective channel (VDAC (G)) has not been detected upon treatment with different concentrations of aripiprazole and/or Genz. In addition, aripiprazole/Genz-treated cells did not show marked alterations in the expression of ER-stress proteins PERK and Ire1α (B, western blot, H and I, quantification). All samples were from the same experiment and blots were processed in parallel, n=3 for each condition; graphs show mean values ± SD. Significances calculated by an one-way Anova-test are: *, $p \leq 0.05$; **, $p < 0.01$; ***, $p < 0.001$; ****, $p<0.0001$

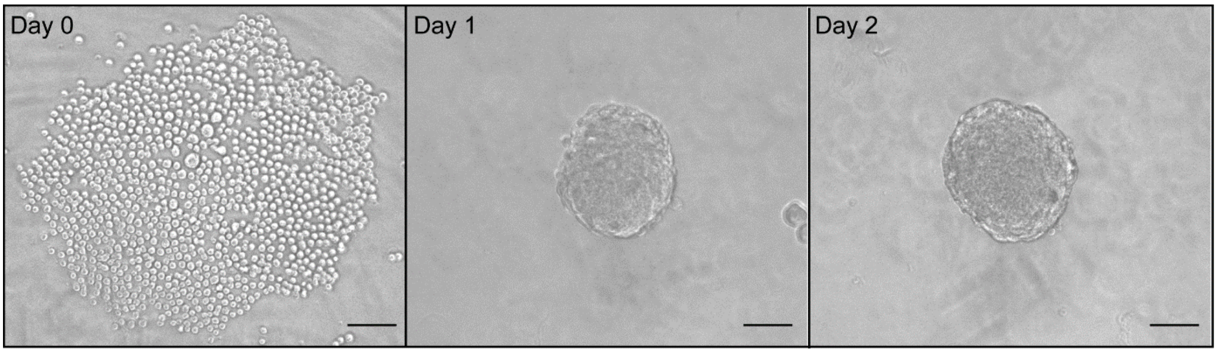

Supporting Figure S4

Formation of hepatocellular tumor microspheres. 10^3 cells of the respective cancer cells were seeded into ultra-low attachment plates (Day 0). Microspheres form automatically after 24h (Day 1). Spheroid size increased already one day later (Day 2).
